# Supplementary material for: Patterns of Intron Gain and Loss in Fungi
Source: PLoS Biol. 2004 Nov 30;2(12):e422. doi: 10.1371/journal.pbio.0020422 (PMC532390; doi:10.1371/journal.pbio.0020422)
Supplement: Table S1 — Also available at http://genes.mit.edu/NielsenEtAl/. (4.3 MB ZIP). [file pbio.0020422.st001.zip › NielsenEtAl/html/763.html]

AN2867.1.NCU10058.1.MG04495.1.FG00387.1


```
 CLUSTAL W (1.82) Multiple Sequence Alignments - Introns Inserted


Sequence 1: NCU10058.1	554 aa
Sequence 2: MG04495.1	554 aa
Sequence 3: FG00387.1	553 aa
Sequence 4: AN2867.1	556 aa
Alignment Length: 556 aa
Number Identitical Residues: 372 aa
Alignment Score (without introns) 16576


MG04495.1 	MEFKTVEFKSFTDQKPGT2SGLRKKVTVFQQPHYSESFVTSILLSIPEGADG1AFLVIGG
NCU10058.1	MEVQTVEFKPFTDQKPGT2SGLRKKVTVFQQPHYSEAFITSILLSIPEGAEG1AFLVIGG
FG00387.1 	MGVQTVEFKPFQDQKPGT2SGLRKKVVVFQQPHYSEAFITSILLSIPEGVEG1SNLVIGG
AN2867.1  	MSVQTVSIQPFGDQKPGT2SGLRKKVKIFQQENYTESFLTSIFLSIPEGAQD~AFLVIGG
          	* .:**.::.* ****** ******* :*** :*:*:*:***:******.:. : *****

MG04495.1 	DGRFYNPEVIQLIAKIGLAYGVKKLLIGQNGILSTPAASHVIRKRKATGGILLTASHNPG
NCU10058.1	DGRFWNPEVIQLIAKIGAAYGVKKLLIGQDGILSTPAASHVIRKRKATGGILLTASHNPG
FG00387.1 	DGRYFNPEAIQLIAKIGAAYGVKKLIIGQNGILSTPAASHVIRIRKATGGILLTASHNPG
AN2867.1  	DGRYYNSDVIQKIAKIGAAYGVKKLIVGQNGILSTPAASNLIRKRKATGGILLTASHNPG
          	***::*.:.** ***** *******::**:*********::** ****************

MG04495.1 	G1PKNDFGIKYNLANGGPAPESVTNKIYETTKTLTSYKIASIPDIDITNIGTKTYEGLEV
NCU10058.1	G1PKNDFGIKYNLANGGPAPESVTNKIYEVSKTLTSYKIASIPNIDISTIGTKTYGDLEV
FG00387.1 	G1PKNDFGIKYNLANGGPAPESVTNKIYEFSKTLTSYKIADIPDVDITTVGTQTYGALEV
AN2867.1  	G1PDNDFGIKYNLTNGAPAPEQVTNKIYEVSKSLTSYKYIDLPEVDTTTVGTRSYGPLEV
          	* *.*********:**.****.******* :*:*****  .:*::* :.:**::*  ***

MG04495.1 	EVVDSCEDYTAMLKDIFDFDLIKKFFASNSDFKVLFDGLSGVTGPYGKAIFQEELGLGAE
NCU10058.1	EVVDSTADYVEMLKDIFDFDLIKKFFATHPDFKVLFDGLSGVTGPYGKAIFQQELGLGSE
FG00387.1 	EVIDSTADYVAMLKDIFDFDLIKKFFSSHPDFKVLFDGLHGVTGPYGKAIFEQELGLSN-
AN2867.1  	EVVHSTEDYVSMMKEIFDFDLIRSFLKKHPDFKVLFDGMHGVTGPYGIDIFVNELGLPSS
          	**:.*  **. *:*:*******:.*: .:.********: *******  ** :****  .

MG04495.1 	STQNCEPSPDFNGGHPDPNLVYAKSLVDVVEEKKIPFGAASDGDGDRNMIYGAGAFVSPG
NCU10058.1	STQNCEPSPDFNGGHPDPNLTYAHSLVETVEKNNIPFGAASDGDGDRNMIYGAGAFVSPG
FG00387.1 	STQNCIPSPDFNGGHPDPNLTYAHSLVEVVDKHNIPFGAASDGDGDRNMIYGANAFVSPG
AN2867.1  	STMNCIPKPDFGGGHPDPNLVYAHELVEAVDKNGIHFGAASDGDGDRNMIYGANTFVSPG
          	** ** *.***.********.**:.**:.*::: * *****************.:*****

MG04495.1 	DSLAIIAHHAKLIPYFKKNGVFGLARSMPTSGAVDLVAKKQGLNCYEVPTGWKFFCALFD
NCU10058.1	DSLAIIAHHAQLIPYFKKNGVYGLARSMPTSGAVDLVAKKQGLNCYEVPTGWKFFCALFD
FG00387.1 	DSLAIIAHYAHLIPYFKKNGVNGLARSMPTSGAVDLVAKAQGLDCYEVPTGWKFFCALFD
AN2867.1  	DSLAIIAHHAKLIPWFQKHGVDGLARSMPTSGAVDRVAKAQGLQSYEVPTGWKFFCNLFD
          	********:*:***:*:*:** ************* *** ***:.*********** ***

MG04495.1 	ADKLSICGEESFGTGSNHIREKDGLWAIVAWLNIIAGLGVEHPDVTPSIKQIQLDFWKEY
NCU10058.1	ANKLSICGEESFGTGSNHIREKDGLWAIVAWLNIIAGLGVANPGVAPSIKQIQKDFWAEY
FG00387.1 	AKKLSICGEESFGTGSDHIREKDGLWAVIAWLNIIAGVGIQNPEVTPSIKEIQKEFWTKY
AN2867.1  	NKKMSICGEESFGTGSNHIREKDGVWAIVAWLNVIAGVAEQKPNETPSIASIQAEFWETY
          	 .*:************:*******:**::****:***:.  :*  :*** .** :**  *

MG04495.1 	GRTFFTRYDYEDVDSEGANKLVKTLQEKMAD--SSFVGSKIGD1LTVTEAGDFSYTDLDG
NCU10058.1	GRTFFTRYDYEDVDSEGANKVVGILRDLVAD--PNFVGSKVGD1RTVTEAGDFSYTDLDG
FG00387.1 	GRTFFTRYDYEDVDSEGANKVIGELEKLVAD--SNFVGSTIEG1RKVTKAGNFSYTDLDG
AN2867.1  	GRTFFTRYDYENVDSDGANKLIAALSEKAVDNKSSFVGSTISG~RKVVDSGNFAYTDLDG
          	***********:***:****::  * .  .*....****.: .  .*..:*:*:******

MG04495.1 	SVSKNQGLYVRFSSGSRIVVRLSGTGSSGATIRLYIEQYSDDASTYEKDAQDFLGPEIKF
NCU10058.1	SVSSNQGLYARFSSGSRIVVRLSGTGSSGATIRLYIEQHSTDPATYDMDAQDFLAPEIKM
FG00387.1 	SVASKQGLYAGFSSGSRIVVRLSGTGSSGATIRLYIEQHTSDPSKYELDAQDFLKEEVKF
AN2867.1  	SVTKNQGLYVKFDDGSRLVVRLSGTGSSGATIRLYVEKYEGDKSKYQMATQDYLKDNVGL
          	**:.:****. *..***:*****************:*::  * :.*:  :**:*  :: :

MG04495.1 	ATELLKFKEFVGRDEPDVKT
NCU10058.1	ATELLKFKEFVGRDEPDVKT
FG00387.1 	ATELLKFKEHVGRDEPDVKT
AN2867.1  	ALELLKFKEFVGREEPDVKT
          	* *******.***:******
```
